# The longitudinal association between Perceived Stress, PTSD Symptoms, and Post-Traumatic Growth during the COVID-19 Pandemic: the role of coping strategies and psychological inflexibility

**DOI:** 10.1007/s12144-022-03502-3

**Published:** 2022-07-26

**Authors:** Francesco Bruno, Francesca Vozzo, Domenico Arcuri, Raffaella Maressa, Elisabetta La Cava, Antonio Malvaso, Chloe Lau, Francesca Chiesi

**Affiliations:** 1 Regional Neurogenetic Centre (CRN), Department of Primary Care, ASP Catanzaro, Viale A. Perugini, Lamezia Terme, CZ Italy; 2 Association for Neurogenetic Research (ARN), Lamezia Terme, CZ Italy; 3 Academy of Cognitive Behavioral Sciences of Calabria (ASCoC), Lamezia Terme, Italy; 4 Department of Medical and Surgical Sciences, Magna Graecia University of Catanzaro, Catanzaro, Italy; 5 Neurology Unit, IRCCS San Raffaele Scientific Institute, Milan, Italy; 6 Department of Psychology, Western University, London, ON Canada; 7 Department of Neuroscience, Psychology, Drug, and Child’s Health (NEUROFARBA), Section of Psychology, University of Florence, Via S.Salvi 12, 50135 Florence, Italy

**Keywords:** Perceived stress, Post-Traumatic Stress Disorder Symptoms (PTSD), Post-Traumatic Growth, COVID-19 Pandemic, Coping strategies, Orientation to the problem, Avoidance strategies, Positive attitudes, Psychological inflexibility

## Abstract

The present study investigates the mediating roles of psychological inflexibility and differential coping strategies on perceived stress and post-traumatic symptoms and growth in the context of COVID-19. Study one recruited 662 participants (54.8% women; M_age_ = 40.64 years, SD = 13.04) who completed a cross-sectional questionnaire. It was proposed that orientation to the problem, avoidance strategies, psychological inflexibility, and positive attitude were mediators for the positive association between perceived stress and PTSD symptoms. The fit indices for the path model were excellent: CFI = 0.977, TLI = 0.950, RMSEA = 0.057 [90%CI = 0.043–0.081], and SRMS = 0.042. Gender and stressful events encountered had indirect effects on the endogenous variables. In study two, 128 participants (57.8% women; M_age_ = 42.30, SD = 12.08) were assessed for post-traumatic growth one year later. Psychological inflexibility and orientation acted as mediators between perceived stress and PTSD symptoms. Furthermore, a novel path model was constructed in which psychological inflexibility and orientation to the problem as mediators for perceived stress and PTSD symptoms. The indices for the path model were excellent: CFI = 0.99, TLI = 0.97, RMSEA = 0.055 [90%CI = 0.001–0.144], and SRMS = 0.49. Furthermore, PTSD symptoms, psychological inflexibility, and orientation to the problem predicted post-traumatic growth. Specifically, both orientation to the problem (β = .06 [90%CI: .01;.13]) and psychological inflexibility (β = .14 [90%CI: .08;.26]) had an indirect effect on post-traumatic growth. Overall, these results significantly contribute to the literature as orientation to the problem positively predicted PTSD symptoms and post-traumatic growth one year later while psychological inflexibility predicted PTSD symptoms and less post-traumatic growth one year later. These results underline the importance of assessing both symptomology and psychological growth to determine adaptive coping strategies in specific contexts.

## Introduction

The Coronavirus Disease 2019 (COVID-19) Pandemic represents an emergency for international public health both from a physical and mental standpoint (Gan & Fu, 2022; Ng et al., 2020; Vindegaard & Benros, 2020). The sudden public health crisis of COVID-19, the social restrictions that limit the spread of infections, and the fear of potentially fatal consequences that could affect individuals and their loved ones produce a severely distressing stressor for many individuals (Bridgland et al., 2021; Flesia et al., 2020). In response to the exposure to a stressor that readily or perceptually threatens homeostasis, individuals could generate a collective of physiological, cognitive, emotional, or behavioral responses, collectively known as *stress*, with the goal of re-establishing homeostasis (Chrousos, 2009; Flesia et al., 2020). Depending on the type, timing, and severity of exposure to a stressor, the stress response could be acute or severe/chronic with short- and long-term consequences on physical and psychological health, respectively (Musazzi et al., 2017). The exposure to a severe or traumatic stressor could also determine the onset of Post-Traumatic Stress Disorder (PTSD) Symptoms (Bridgland et al., 2021). Based on DSM–5 criteria, PTSD Symptoms can be categorized into four clusters: i) *intrusion/re-experiencing* symptoms (e.g., intrusive memories and nightmares of the trauma); ii) *avoidance* symptoms (e.g., avoidance of distressing memories and thoughts about the trauma); iii) *negative cognitions* and *mood* (e.g., inability to remember important aspects of the trauma and persistence negative emotional state, like fear, horror, anger, guilt, or shame); iv) and *alterations in arousal and reactivity* (e.g., hypervigilance, irritability and angry outbursts with little or no provocation) (*American Psychiatric Association*, APA, 2013).

Several studies reported a simultaneously high percentage of both perceived stress and PTSD symptoms during the first wave of COVID-19 Pandemic (Krishnamoorthy et al., 2020; Rossi et al., 2020; Salari et al., 2020). Specifically, Ikizer et al. (2021) found that higher levels of perceived stress predicted PTSD Symptoms and financial stressors, social media use, and time spent in the home were associated with higher post-traumatic stress. However, no studies, to our knowledge, have analyzed whether certain mediators may enhance or reduce this association. Moreover, longitudinal studies are needed to investigate post-traumatic growth after the onset of PTSD symptoms.

### COVID-19 stressful events, perceived stress and PTSD symptoms

A systematic review and meta-analysis on the global general population reported almost half the general public’s mental health symptoms were negatively affected by COVID-19, with a prevalence of 34% reporting perceived stress, 34% reporting psychological distress, and 27% reporting PTSD Symptoms during the first wave of COVID-19 Pandemic (Krishnamoorthy et al., 2020). Interestingly, perceived stress prevalence was higher amongst the general population (36%) compared to healthcare workers (33%), while stress amongst COVID-19 patients was not reported. In Italy, a cross-sectional study conducted on 18,147 adults during the mandatory lockdown estimated a rate of 37% for PTSD Symptoms and of 22.9% for perceived stress. These outcomes were positively associated with female gender, suggesting that women may be at heightened risk for psychological distress (Rossi et al., 2020). Besides gender, perceived stress is associated with the onset of COVID-19 stressful events. Not surprisingly, some COVID-19 stressful events, such as contracting COVID-19 and having a loved one inflected or deceased due to COVID-19, heightened the levels of perceived stress (Mousavi et al., 2020; Rossi et al., 2020).

To date, only two studies have examined the association between perceived stress and PTSD symptoms in two different stressful situations: civilian exposition to ongoing terrorist attacks (Besser et al., 2009) and oral cancer (Zhang et al., 2021). Both studies have found that PTSD symptoms were positively associated with perceived stress (Besser et al., 2009; Zhang et al., 2021). In the context of COVID-19 Pandemic, Ikizer et al. (2021) found similar results in a sample of 685 Turkish participants. The authors hypothesized that limited access to psychological and material resources to cope with an unexpected aversive event contributed to this association. As such, it is important to emphasize the relevance of examining this association in other countries to investigate the role of the psychological resources, coping, and psychological inflexibility in coping with the onset of COVID-19 stressful events.

### Perceived stress, PTSD symptoms and coping strategies

Although similar events may affect many different individuals, individual responses may vary depending on the coping strategy of choice (Folkman, 2010). Coping strategies refer to *“thoughts and behaviors that people use to manage the internal and external demands of situations that are appraised as stressful”* (Folkman & Moskowitz, 2004, p. 746). According to the model proposed by Foà et al., (2015), individuals mainly use five different coping strategies: i) *orientation to the problem* (i.e., the tendency to use strategies which aim to dominate events through cognitive processes, suppression activities, planning and search for information, underestimating the activities which help the management of stressful situations); ii) *positive attitude* (i.e., the tendency to proactively accept events, containment and positive reinterpretation of events, transforming them into opportunities for growth); iii) *transcendent orientation* (i.e., the tendency to rely on aspects and activities related to religion and spirituality); iv) *social support* (i.e., the tendency to implement requests for understanding, information and emotional support in their social/professional network; v) *avoidance strategies* (i.e., the tendency to ignore and deny the threat of stressful events, through activities which divert attention from the problem using humor and behavioral and mental detachment; Foà et al., 2015). Several studies examined the relationship between perceived stress and endorsement of coping strategy usage (Cai et al., 2020; Khalid et al., 2016; Phua et al., 2005). Differential endorsement of these coping strategies have also been associated with PTSD symptoms (Cofini et al., 2015; Grosso et al., 2014; Gutner et al., 2006; Ozer et al., 2003). In particular, maladaptive coping strategies, such as avoidance strategies, are associated with greater PTSD severity (Clohessy & Ehlers, 1999; Hooberman et al., 2010) whereas the functional problem- or emotion-focused coping strategies are protective and associated with lower levels of PTSD (Linley & Joseph, 2004; Ozer et al., 2003). However, some studies question the dichotomy “adaptive/maladaptive coping strategies,” suggesting the importance of context in which coping occurs (Fischer et al., 2021; Moritz et al., 2016). As Fischer et al. (2021) argue, the functionality of coping strategies may depend on the context in which they are executed, as they are influenced by contextual factors.

For example, it is notable a relationship between emotions and some coping strategies (Pachter et al., 2013). Indeed, regarding the COVID-19 Pandemic context, several studies have confirmed the mediating role of emotions on coping strategies (Gan & Fu, 2022; Gan et al., 2021). On the other hand, it may be important to examine coping strategies from a different point of view and to analyze whether they may be considered adaptive or maladaptive as mediators of the relationship between perceived stress and PTSD symptoms in the context of the COVID-19 Pandemic.

### Perceived stress, PTSD symptoms and psychological inflexibility

Psychological inflexibility refers to the rigid attempts to control psychological responses of discomfort (e.g., thoughts, affect), to the detriment of values-guided actions (Bond et al., 2011; Tavakoli et al., 2019). In other words, when managing distressing emotions and thoughts, individuals with high levels of psychological inflexibility tend to avoid, minimize, or control their experiences and act inconsistently with their values (Tavakoli et al., 2019). According to Acceptance and Commitment Therapy (ACT; Hayes et al., 2011), psychological inflexibility represents a transdiagnostic process across psychological disorders (Levin et al., 2014). Previous studies reported a positive correlation between perceived stress and psychological inflexibility (Arslan et al., 2021; Tavakoli et al., 2019). Psychological inflexibility was related with the onset and severity of PTSD Symptoms in several populations such as veterans (e.g., Brockman et al., 2016; Crabtree et al., 2021; Meyer et al., 2019), war survivors (Kashdan et al., 2009), community members (Bardeen & Fergus, 2016), and inpatient adolescents (Schramm et al., 2020). During the Italian lockdown caused by COVID-19 Pandemic, Pakenham et al. (2020) found that psychological inflexibility exacerbates the adverse effects of several COVID-19 stressful events (e.g., having contracted the COVID-19, having a family members infected, hospitalized, or deceased due to COVID-19) on mental health (i.e., peritraumatic distress, anxiety, and depression). Moreover, Pakenham et al. (2020) explored four psychological flexibility sub-processes of self-as context, defusion, values, and committed action that mitigated the negative impacts of COVID-19 risk factors.

Dawson and Golijani-Moghaddam (2020) suggest that psychological inflexibility and coping strategies are two different, yet intertwined, constructs. Indeed, the level of psychological flexibility influences the selection of coping strategies. For example, the presence of high psychological inflexibility determines the implementation of avoidance strategies (Dawson & Golijani-Moghaddam, 2020). In addition, psychological inflexibility has been successfully tested as a mediator in the relationship between different constructs such as COVID-19 stress and psychological distress (i.e., depression, anxiety, and somatization) (Arslan et al., 2021), perceived stress, anxiety, and depression (Huang et al., 2021), intolerance of uncertainty, and PTSD symptoms (Kennedy et al., 2021). One limitation is that the potential mediating role of psychological inflexibility in the relationship between perceived stress and PTSD symptoms in any stressful situation, as well as in the context of COVID-19 Pandemic, has not been examined. These findings underline the need for further research on this topic.

### PTSD symptoms and post-traumatic growth

Stressful and difficult life events can lead the individual to positive changes, according to the evidence that struggling with highly challenging life circumstances can involve post-traumatic growth (Tedeschi & Calhoun, 2004). Several studies considered post-traumatic growth, in response to a stressor, as a positive reinterpretation process distinguished by a greater sense of perceived strength, higher quality interpersonal relationships, and a greater appreciation for life (Koliouli & Canellopoulos, 2021; Tedeschi & Calhoun, 2004). The relationship between perceived stress and post-traumatic growth has been analyzed in different contexts, such as breast cancer (Groarke et al., 2017; Yeung & Lu, 2018), earthquakes (Taku et al., 2015) and work satisfaction (Xu & Wu, 2014). In particular, Coroiu et al. (2016) found that moderate perceived stress levels may be linked to higher post-traumatic growth, which may reflect more adaptive outcomes. Regarding the relationship between post-traumatic growth and PTSD symptoms, higher levels of distress is not reflective of an absence of psychological growth and maturation (Solomon & Dekel, 2007). Solomon and Dekel (2007) indicated although an individual may grow and experience positive changes after the traumatic event, it does not undo the suffering of the event. Instead, their findings supported a quadratic function in the association between PTSD symptoms and growth, suggesting that moderate levels of symptoms may maximize growth optimally. During the COVID-19 crisis, optimism was found to decrease intrusive thoughts and avoidance behaviors and may enhance posttraumatic growth (Koliouli & Canellopoulos, 2021). However, the aforementioned cross-sectional studies forbid any causal interpretations. Thus, the present study aims to analyze these associations in a longitudinal perspective.

## General aims

Under the light of these premises, our general aims are: i) to understand the causal relationship between COVID-19 stressful events, perceived stress, coping strategies, psychological inflexibility, and PTSD Symptoms during the first wave of COVID-19 (**Study 1**); ii) to investigate the persistence of these associations one-year later and its relationship with Post-Traumatic Growth (**Study 2).**

## Study 1

As mentioned above, the general aim of study 1 was to characterize the temporal association between COVID-19 stressful Events, perceived stress, coping strategies, psychological inflexibility, and PTSD Symptoms during the first wave of COVID-19 Pandemic, taking into account Gender and the COVID-19 Stressful Events.

In that time (from 9^th^ march to 3^th^ June 2020), to prevent and contain the spread of the infection, the Government of the Italian Republic adopted mandatory lockdown measures on the entire national territory (Rossi et al., 2020). Mandatory lockdown measures included: ban on moving in and out of the regions except for work requirements or situations of necessity (e.g., medical emergencies). Subjects with symptoms of respiratory infection and fever were required to quarantine at home and avoid social contacts. Infected individuals were subjected to mandatory quarantine. Events and sports competitions were suspended. In-person educational events and classes were suspended, replaced by distance learning. Civil and religious ceremonies were not allowed while museums and other cultural institutions and places were closed. Gyms, swimming pools, cultural centers, social centers and recreational centers were also closed. (https://www.governo.it/it/coronavirus; Motta Zanin et al., 2020).

**Fig. 1:**
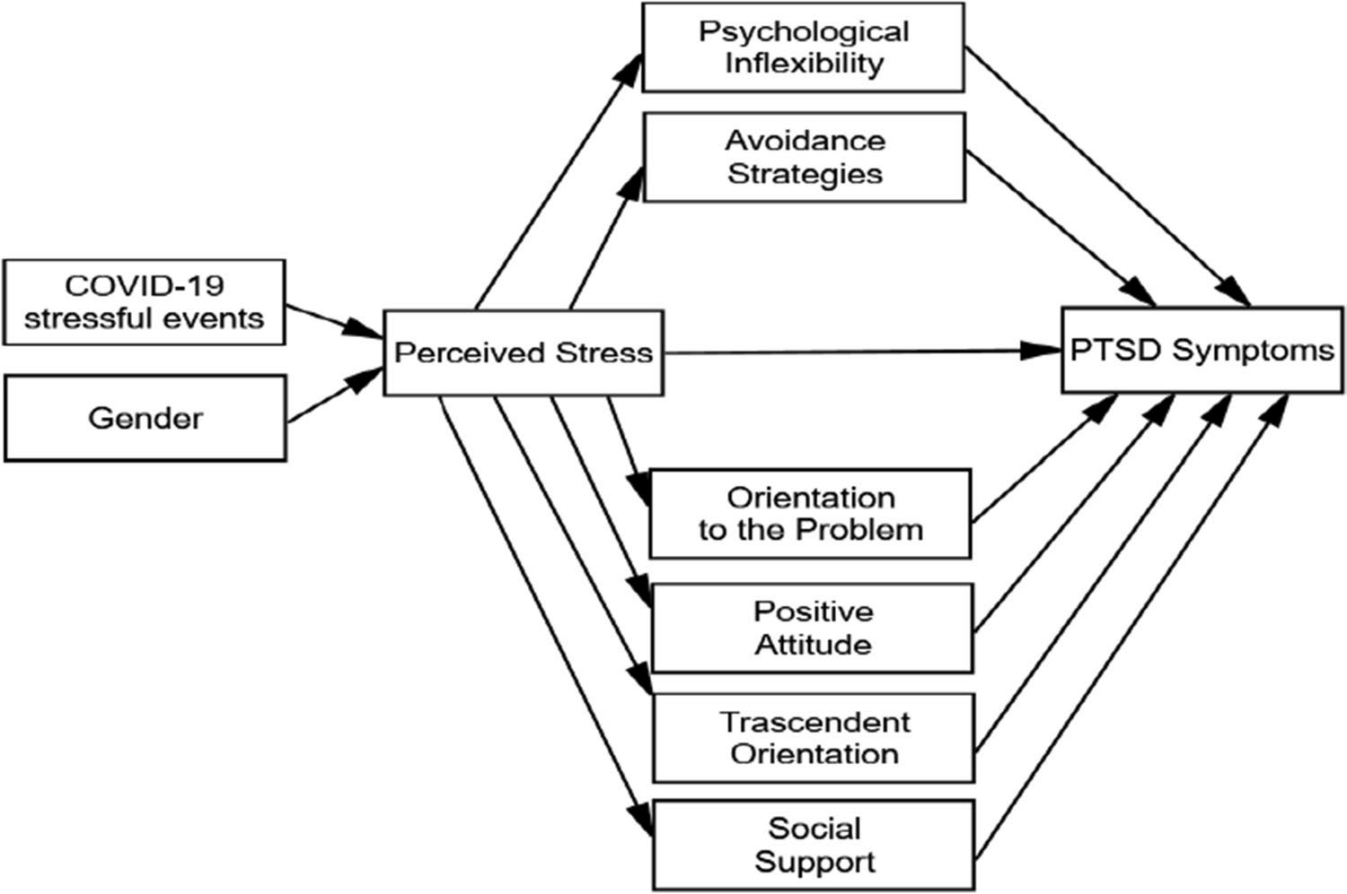
The initial hypothesized path model illustrating direct and indirect effects and causal paths linking exogenous and endogenous variables with PTSD Symptoms

Based on the literature above discussed, the following hypotheses (H1-H7) were formulated (Fig. 1):

- **H1.** Onset of COVID-19 stressful events were positively related with perceived stress.
- **H2.** Female gender was positively related with perceived stress;
- **H3.** Perceived stress was positively related to PTSD symptoms;
- **H4.** Perceived stress was related to coping strategies;
- **H5.** Perceived stress was positively related to psychological inflexibility;
- **H6**: Maladaptive coping strategies (i.e., avoidance strategies) and psychological inflexibility were positively related with PTSD symptoms;
- **H7.** Adaptive coping strategies (i.e., orientation to the problem, positive attitude, transcendent orientation, social support) were negatively related to PTSD symptoms.

## Method

### Measures

#### COVID-19 stressful events

Stressful events related to COVID-19 were analyzed with the following questions: 1) “Have you contracted the flu during the COVID-19 Pandemic?” (Yes/No), 2) “Have you been tested for COVID-19?” (Yes/No), 3) “Have you contracted COVID-19?” (yes/no), 4) “Has anyone in your family contracted COVID-19?” (Yes/No), 5) “Have you been bereaved due to COVID-19?” (Yes/No). A total score was obtained summing answers (Yes = 1) with higher score indicating greater frequency of stressful events experienced.

#### Perceived Stress Scale (PSS-10)

Perceived stress was assessed through the Italian version of the Perceived Stress Scale (PSS-10; Mondo et al., 2019). Each of the PSS 10-items is rated on a 4-point Likert scale ranging from 0 (Never) to 4 (Very often). Higher scores indicate higher levels of Perceived Stress in response to stressful situations that occurred in the last 4 weeks. In our sample, internal consistency was α = 0.87.

#### Acceptance and Action Questionnaire-II (AAQ-II)

Psychological inflexibility was evaluated through the Italian version of Acceptance and Action Questionnaire-II (AAQ-II; Pennato et al., 2013). The 10 items are rated on a 7-points Likert scale ranging from 1 (It is never true for me) to 7 (It is always true for me). Higher scores indicate higher psychological inflexibility. In our sample, internal consistency was α = 0.89.

#### Coping Orientation to the Problems Experienced (COPE-NVI-25)

Coping strategies were assessed using the Italian version of Coping Orientation to the Problems Experienced (COPE-NVI-25; Foà et al., 2015). The 25 items are rated on a 6-points Likert scale ranging from 1 (I usually don't do this at all) to 6 (I usually do this a lot). They allow to measure the frequency with which are used five independent coping strategies: avoidance strategies (COPE-AS, 5 items), orientation to the problem (COPE-OP, 5 items), positive attitude (COPE-PA, 6 items), transcendent orientation (COPE-TO, 4 items), social support (COPE-SS, 5 items). In our sample, Cronbach's internal consistency αs were 0.64, 0.72, 0.71, 0.96, and 0.83, respectively.

#### Impact of Event Scale-Revised (IES-R)

PTSD Symptoms were evaluated using the Italian version of Impact of Event Scale-Revised (IES-R; Pietrantonio et al., 2003). The 22 items are rated on a 5-point Likert scale ranging from 0 (Not at all) to 4 (Extremely). Higher scores indicate higher levels of PTSD Symptoms experienced in the last 7 days. In our sample, internal consistency was α = 0.93.

### Design and procedure

As part of a large study evaluating the psychological impact of COVID-19 on the Italian general population, all subjects meeting the following criteria based on self-report were included in this sample: 1. Italian citizens living in Italy during the COVID-19 mandatory lockdown; 2. Adults (i.e., age 18 and older); 3. No mental health diagnoses prior to COVID-19 pandemic; 4. Not currently taking psychiatric medication; 5. Not recently experienced any other potentially traumatic or stressful event in addition to COVID-19; 6. All questions administered in the questionnaires were completed. The study was a web-based survey designed for involving participants of all Italian Regions. The survey was developed using Google Forms® and was distributed through social networking sites such as Facebook, WhatsApp, and Instagram—using sponsored social network advertisement together with a snowball recruiting technique—during the period of Italian mandatory lockdown (between 18^th^ April and 3^th^ June 2020). Participants were informed that their participation was voluntary, their responses would be anonymous, and they could withdraw from the survey at any time if they did not want to continue. All participants signed an on-line informed consent and did not receive an incentive. The research protocol was approved by the Ethical Committee of Regione Calabria—Area Centrale (Catanzaro, Italy).

### Analysis strategy

All analyses were conducted on SPSS and its extension Amos (version 27.0). Preliminarily, descriptive and Spearman’s rho correlations were computed for the variables included in the initial hypothesized model (Fig. 1). Specifically, the obtained correlations—among two exogenous variables (Gender and COVID-19 Stressful Events) and eight endogenous variables (PSS-10, COPE-OP, COPE-PA, COPE-TO, COPE-SS*,* COPE-AS, AAQ-II, and IES-R)- allowed to identify which variables had to be included in the path analysis.

Then, path analysis was carried out to estimate the magnitude and significance of hypothesized causal connections among exogenous and endogenous variables. We used the Maximum Likelihood Estimation (MLE) method to estimate the regression/path coefficients and the relative confidence intervals were computed with bootstrap sampling (number of bootstrap samples = 500). Model fit was assessed using the following criteria: Comparative Fit Index (CFI) ≥ 0.95, Tucker-Lewis Index (TLI) ≥ 0.95, the Root Mean Square Error of Approximation (RMSEA) and the Standardized Root Mean Square Residual (SRMR) ≤ 0.06 (Schreiber et al., 2006).

### Sample size definition

Suggested sample sizes based on ML estimation is between 200 and 500, depending on the complexity of the model (Jackson, 2001) and the presence of binary variables, such as gender in our model (Bandalos, 2014). Additionally, Wolf et al. (2013) suggested that, with respect to the magnitude of regressive effects in the model, effects that are very weak or very strong may require larger samples, and this effect is more pronounced in models with very weak effects. Since the interplay between the variables of the current study has not been investigated before, we deemed adequate to use a large sample (about *N* = 650) to be able to obtain unbiased estimations for all the possible strengths of the observed relationships. Finally, because we planned a retest after one year, we preferred to have an initial large sample in Study 1, expecting a significant dropout in Study 2.

## Results

### Participants

The sample employed for this study was composed by 662 (54.8% women) with a mean age of 40.64 years, ranging from 18 to 76 years (*SD* = 13.04). 54.3% of the respondents completed college, 40.3% had achieved higher education, and 5.4% had less than a high school education. 27.9% of participants were single, 64% were in a relationship, and 8.1% were divorced or separated or widowed. Finally, respondents’ place of stay in Italy, classified as North, Center, and South of Italy, were 13.6%, 60.7%, and 25.7%, respectively.

### Descriptives and correlations

**Fig. 2:**
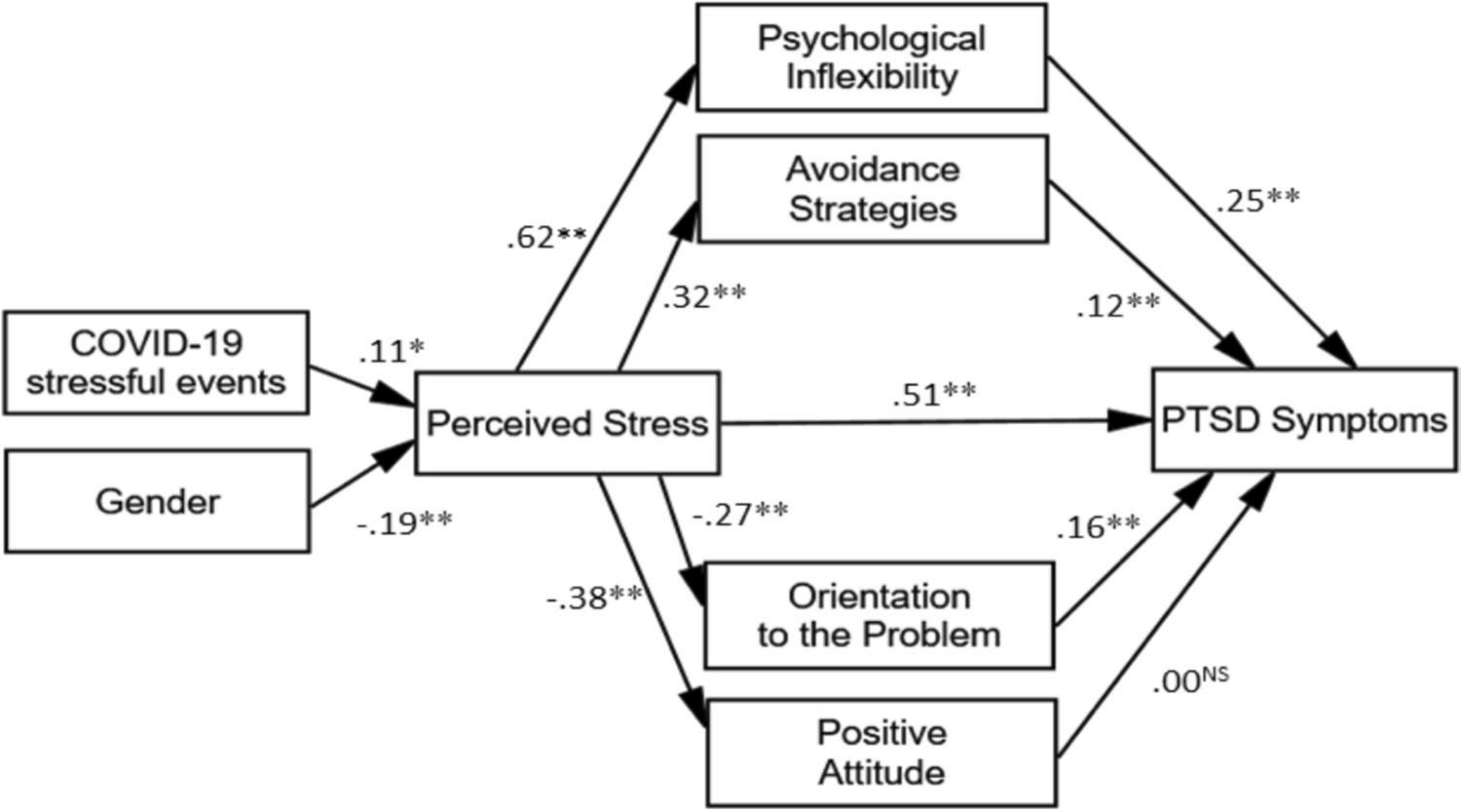
The path model illustrates direct and indirect effects and causal paths linking variables with PTSD Symptoms. *Note.* Values on the line = standardized path coefficient. * *p* < 0.01 and ** *p* < 0.001, NS = non-significant. Gender: F = 1 and M = 2. PSS-10 = Perceived Stress Scale, AAQ-II = Acceptance and Action Questionnaire-II, COPE-AS = avoidance strategies, COPE-OP = orientation to the problem, COPE-PA = positive attitude, IES-R = Impact of Event Scale-Revised., PTSD = post-traumatic stress

The COVID-19 stressful events score indicated that 10% (*n* = 65) of the sample were directly or very closely hit by the virus. The correlations among the measured variables (Table 1) showed that all the expected relationships were significant apart from two out of five coping strategies. Transcendent orientation and social support were correlated with the other coping strategies, but not with the other variables in the hypothesized model. Whereas the strength of the correlation was low, gender correlated both with perceived stress (*r* = 0.20) and PTSD symptoms (*r* = 0.12). The expected correlation between perceived stress and COVID-19 stressful events was very low (*r* = 0.11). Perceived Stress was correlated (effect sizes were from medium to large) with avoidance strategies (*r* = *0.3*2), orientation to the problem and positive attitude (*r* = -0.27 and *r* = -0.38, respectively), and psychological inflexibility and PTSD symptoms (*r* = 0.62 and *r* = 0.67, respectively). Psychological inflexibility and avoidance strategies were correlated with PTSD Symptoms (*r* = 0.58 and *r* = 0.36, respectively). Finally, PTSD symptoms were slightly correlated with orientation to the problem and positive attitude (*r* = -0.09 and *r* = -0.23, respectively). Starting from this pattern of correlations, we defined the causal model to test with path analysis (Fig. 2).

**Table 1:** Means, standard deviations, Cronbach alphas, and bivariate correlates between the variables of the study

	*M*	*SD*	(1)	(2)	(3)	(4)	(5)	(6)	(7)	(8)	(9)	(10)
(1) Gender	-	-	*-*									
(2) COVID-19 Stressful Events	0.29	0.76	-0.04	*-*								
(3) PSS-10	1.89	0.84	-0.20^**^	0.11^*^	*0.87*							
(4) AAQ-II	2.95	1.21	-0.06	0.06	0.62^**^	*0.89*						
(5) COPE-AS	1.67	0.53	0.04	0.01	0.32^**^	0.48^**^	*0.64*					
(6) COPE-OP	3.11	0.54	0.03	-0.04	-0.27^**^	-0.31^**^	-0.28^**^	*0.72*				
(7) COPE-PA	3.07	0.53	0.05	-0.04	-0.38^**^	-0.37^**^	-0.16^*^	0.55^**^	*0.71*			
(8) COPE-TO	1.65	0.93	-0.04	0.03	-0.03	-0.01	0.09^*^	0.16^**^	0.09^*^	*0.96*		
(9) COPE-SS	2.67	0.66	-0.18^**^	0.04	-0.03	-0.08^*^	0.05	0.47^**^	-0.20^**^	0.17^**^	*0.83*	
(10) IES-R	1.43	0.80	-0.12^**^	0.09^*^	0.67^**^	0.58^**^	0.36^**^	-0.09^*^	-0.23^**^	0.07	0.01	*0.93*

*N* = 662. Cronbach alphas in diagonal are in *italics*. * *p* < 0.01 and ** *p* < 0.001 (adjusted level of significance to adjust for Type 1 error). Gender: F = 1 and M = 2. PSS-10 = Perceived Stress Scale, AAQ-II = Acceptance and Action Questionnaire-II, COPE-AS = avoidance strategies, COPE-OP = orientation to the problem, COPE-PA = positive attitude, COPE-TO = transcendent orientation, COPE-SS = social support, IES-R = Impact of Event Scale-Revised

### Path analysis

The fit indices for the model were CFI = 0.977, TLI = 0.950, RMSEA = 0.057 [90%CI = 0.043–0.081], and SRMS = 0.042, indicating an excellent fit. Estimates of the path coefficients and the results of significance testing are presented in Table 2 and Fig. 2. Of the eleven path coefficients (corresponding to the seven hypotheses), ten were found to be statistically significant (Table 2). As for the exogenous variables, the standardized regression coefficient for COVID-19 stressful events was 0.11 indicating that 11% of the variance in perceived stress can be explained by this variable, while gender predicted 19% of perceived stress variability. In turn, perceived stress explained orientation to the problem (27%), positive attitude (38%), and avoidance strategies (32%). Additionally, perceived stress explained a large part (62%) of psychological inflexibility. Finally, orientation to the problem, avoidance strategies, and psychological inflexibility explained 16%, 12%, and 25% of PTSD symptoms, respectively.

**Table 2:** Results from testing the hypotheses using path analysis parameter estimates

*Hypotheses*	*Path*	*Path coefficient [90%CI]*	*t*	*P*
H1	COVID-19 Stressful Events → PSS-10	0.105 [0.042;0.177]	2.767	0.006
H2	Gender → PSS-10	-0.192 [-0.256;-0.127]	-5.063	< 0.001
H3	PSS-10 → COPE-AS	0.316 [0.259;-0.367]	8.553	< 0.001
	PSS-10 → COPE-OP	-0.266 [-0.323;-0.200]	-7.091	< 0.001
	PSS-10 → COPE-PA	-0.384 [-0.440;-0.325]	-10.677	< 0.001
H4	PSS-10 → AAQ-II	0.619 [0.572-;0.656]	20.278	< 0.001
H5	PSS-10 → IES-R	0.514 [0.461;0.563]	14.700	< 0.001
H6	AAQ-II → IES-R	0.250 [0.187;0.317]	6.691	< 0.001
	COPE-AS → IES-R	0.120 [0.066;0.168]	3.943	< 0.001
H7	COPE-OP → IES-R	0.160 [0.099;0.217]	4.976	< 0.001
	COPE-PA → IES-R	*-0.004 [-0.066;0.055]*	*-0.115*	*0.902*

Gender: F = 1 and M = 2. PSS-10 = Perceived Stress Scale, AAQ-II = Acceptance and Action Questionnaire-II, COPE-AS = avoidance strategies, COPE-OP = orientation to the problem, COPE-PA = positive attitude, IES-R = Impact of Event Scale-Revised

Gender had indirect effects on all the endogenous variables included in the model. Indeed, bootstrap confidence intervals for indirect standardized effects did not include a zero, suggesting that all the indirect effects tested are supported. Specifically, gender had an indirect effect on positive attitude (β = 0.074 [90%CI: 0.035;0.076]), on orientation to the problem (β = 0.051 [90%CI: 0.050;0.103]), on avoidance strategies (β = -0.061[90% CI: -0.084;-0.042], on psychological inflexibility β = -0.119 [90%CI: -0.161;-0.078], and on PTSD Symptoms (β = -0.128 [90%CI: -0.173;-0.085]). COVID-19 stressful events also have indirect effects on all the endogenous variables included in the model. Specifically, they had an indirect effect on positive attitude (β = -0.040[90%CI: -0.071;-0.011]), orientation to the problem (β =—0.028 [90%CI: -0.052;-0.009]), avoidance strategies (β = 0.033 [90%CI: 0.014;0.058], psychological inflexibility β = 0.065 [90%CI:0.027;0.111], and PTSD symptoms (β = 0.070 [90%CI: 0.026;0.118]). Finally, perceived stress had a significant indirect effect on PTSD symptoms (β = 0.152 [90%CI: 0.111;0.193]).

## Discussion

Numerous findings were portrayed with the correlational matrix and path analysis model. Transcendent orientation and social support coping styles were not correlated with perceived stress. Previous findings showed that stress mediated the positive correlation between social support and general mental health (Bovier et al., 2004) and stress may interact with social support for positive psychosocial outcomes (Chao, 2012). It is important to note that the data were collected during the height of the COVID-19 pandemic and the role of these coping strategies may differ based on the presenting concern at the time. Furthermore, psychological inflexibility, avoidance, orientation to the problem, and positive attitude mediated the association between stress and PTSD symptoms. These results suggest that all these psychological constructs would be appropriate as treatment targets. Given the strong association between psychological inflexibility and stress, results suggest targeting psychological inflexibility for psychosocial interventions (Meyer et al., 2019). Given that data were collected at the height of the pandemic, these results suggest that coping strategies may be dependent on situational variables. For example, if individuals were living with roommates and family, they are likely surrounded by others and may feel supported, thus negating the role of social support. Moreover, orientation to the problem was negative associated with perceived stress and positively associated with PTSD symptoms. Given that the pandemic produces uncontrollable stressors, a positive perspective (e.g., believing that the pandemic will end eventually) and orientation to the problem (e.g., figuring out how to manage work, meet needs despite restrictions) may be important during this time, but this approach may also be detrimental during uncontrollable circumstances. These results support the notion that differing coping strategies may be adaptive depending on the circumstances. The limitation of study 1 include the cross-sectional nature of the study. Hence, study 2 aims to address this limitation through assessing post-traumatic growth one year later.

## Study 2

Considering the association between perceived stress and PTSD symptoms that we have found during the mandatory lockdown in Italian general population, as well as the role of both adaptive (i.e., orientation to the problem) and maladaptive (i.e., avoidance strategies) coping strategies and psychological inflexibility in predicting the manifestation of PTSD symptoms, we decided to retest participants about 1 year later to evaluate their outcome in terms of post-traumatic growth.

At that time, the government passed the reopening decree in order to gradually return to normality. The “colored regions” system was already in force and it included the classification of the Italian regions in four areas (red, orange, yellow and white) based on the levels of criticality, according to the evolution of the epidemic curve. Particularly, in the red area free movement was not allowed in the municipal district, except for necessity; in the orange and yellow areas it was limited and regulated by a curfew. In the red area there was mandatory distance learning for university, high and middle school, while in the orange and yellow areas it was planned only for high school and university. Convenience stores were opened everywhere, while the activity of other types of shops depended on the area. Cultural activities (e.g., museums, exhibitions, theaters) were closed everywhere. In the white area many restrictions were lifted and most activities were allowed. Meanwhile, the vaccination campaign on citizens was carried out along with the introduction of the Green Pass for a safer circulation and gradually these restrictions were removed (https://www.governo.it/it/coronavirus).

In particular, based on the literature above discussed and the results of study 1 the following hypotheses (H1-H7) were formulated (Fig. 1):

- **H1.** Different COVID-19 Stressful Events were positively related with Perceived Stress.
- **H2.** Female gender was positively related with Perceived Stress.
- **H3-H5.** Perceived Stress was related to coping strategies, psychological inflexibility, and positivity related to PTSD Symptoms.
- **H6**: Maladaptive coping strategies (i.e., avoidance strategies) and psychological inflexibility were positively related with PTSD Symptoms.
- **H7.** Orientation to the problem was negatively related to PTSD Symptoms.
- **H8.** PTSD Symptoms were related to Post-Traumatic Growth.

## Method

### Measures

Participants completed the Italian version of Post-Traumatic Growth Inventory (PTGI; Prati & Pietrantoni, 2014). This scale consisted of 21 items rated on a Likert scale ranging from 0 (I did not experience this change as a result of my crisis) to 5 (I experienced this change to a very great degree as a result of my crisis). Higher score suggested a higher level of Post-Traumatic Growth. In the current sample, internal consistency was α = 0.96.

### Design and procedure

The same participants of the first study were involved in the second study. We have re-contacted the people who had provided the email addresses for free choice with the purpose of taking part in possible following phases of the research. From a total of 320 participants (48.33%) whose email addresses we had, a total of 128 (40%) subjects took part in the study 2. A similar cross-sectional and web-based survey designed for study 1 was adopted for study 2. The survey was developed using the free software Google Forms® and was sent as a link by email. Data were collected between 18^th^ April and 8^th^ June 2021. Participants were informed that their participation was voluntary, that their responses would be anonymous and that they could withdraw from the survey at any time if they did not want to continue. All participants signed an on-line informed consent and did not receive an incentive. Among non-participating subjects, 90.1% of cases were non-responders while in the remaining 9.9% of cases the emails were not delivered.

### Analysis strategy

All analyses were conducted on SPSS and its extension Amos (version 27.0). Preliminarily, descriptives and Spearman rho correlations were computed for the measured variables included in the path model obtained in Study 1 (Fig. 2). Then, the path analysis was replicated using the following variables: Gender and COVID-19 Stressful Events as exogenous variables, PSS-10, COPE-OP, COPE-PA*,* COPE-AS, AAQ-II, and IES-R as endogenous variables with the adding of PTGI as the last outcome variable.

## Results

*Participants.* The follow-up sample was composed by 128 (57.8% women) participants with a mean age of 42.30 years, ranging from 22 to 76 years (SD = 12.08). 40.6% of the respondents completed college, 41.4% had achieved higher education, and 18.0% had less than a high school education. 26.6% of participants were single, 65.6% were in a relationship, and 7.8% were divorced or separated or widowed. Finally, respondents’ place of stay in Italy (classified as North, Center, and South of Italy) were 11.7%, 66.4%, and 21.9%, respectively.

### Descriptives and correlations

The COVID-19 Stressful Events score indicated that 10.9% (*n* = 14) of the sample were directly or very closely hit by the virus. The correlations among the measured variables (Table 3) confirmed the previous ones (Table 1) with minor exceptions. Gender correlated both with perceived stress (*r* = -0.22) but not with PTSD Symptoms (*r* = -0.14). The absence of a correlation between Perceived Stress and COVID-19 Stressful Events was confirmed (*r* = 0.01). As in Study 1, perceived stress was correlated (effect sizes were from medium to large) with avoidance strategies (*r* = *0.2*4), orientation to the problem and positive attitude (*r* = -0.27 and *r* = -0.36, respectively), and psychological inflexibility and PTSD Symptoms (*r* = 0.57 and *r* = 0.58, respectively). Psychological inflexibility and avoidance strategies were correlated with PTSD Symptoms (*r* = 0.56 and *r* = 0.59, respectively), PTSD Symptoms was slightly correlated with positive attitude (*r* = -0.23), and, whereas the size was the same of one reported in Study 1, the correlation with orientation to the problem did not reach the significance level (*r* = -0.09).

**Table 3:** Means, standard deviations, and bivariate correlates between the variables of the study

	*M*	*SD*	(1)	(2)	(3)	(4)	(5)	(6)	(7)	(8)
(1) Gender	*-*	*-*	*-*							
(2) COVID-19 Stressful Events	*0.33*	*0.85*	0.16	*-*						
(3) PSS-10	*1.90*	*0.82*	-0.22^*^	-0.01	*-*					
(4) AAQ-II	*3.02*	*1.29*	-0.06	0.04	0.57^**^	*-*				
(5) COPE-AS	*1.65*	*0.52*	0.01	0.09	0.24^*^	0.59^**^	*-*			
(6) COPE-OP	*3.10*	*0.52*	0.06	0.04	-0.27^*^	-0.35^**^	-0.40^**^	*-*		
(7) COPE-PA	*3.06*	*0.52*	0.12	-0.10	-0.36^**^	-0.40^**^	-0.27^**^	0.60^**^	*-*	
(8) IES-R	*1.45*	*0.75*	-0.14	0.02	0.58^**^	0.56^**^	0.32^**^	-0.09	-0.23^**^	*-*
(9) PTGI	*1.97*	*1.24*	-0.21^**^	0.08	0.04	-0.16	0.01	0.27^*^	0.27^*^	0.19^*^

*N* = 128. Cronbach alphas in diagonal are in *italics*. * *p* < 0.05 and ** *p* < 0.01 (adjusted level of significance to adjust for Type 1 error). Gender: F = 1 and M = 2, PSS-10 = Perceived Stress Scale, AAQ-II = Acceptance and Action Questionnaire-II, COPE-AS = avoidance strategies, COPE-OP = orientation to the problem, COPE-PA = positive attitude, IES-R = Impact of Event Scale-Revised, PTGI = Post-Traumatic Growth Inventory

Finally, post-traumatic growth correlated positively with gender (*r* = 0.22), positive attitude (*r* = 0.27), orientation to the problem (*r* = 0.27), and PTSD Symptoms (*r* = 0.19).

### Path analysis

Starting from the observed pattern of correlations, we tested the causal model of Study 1 adding the variable PTGI. The fit indices for the model were poor (CFI = 0.87, TLI = 0.76, RMSEA = 0.127 [90%CI = 0.091–0.165], and SRMS = 0.11. In particular, COVID-19 stressful events (*r* = 0.07), avoidance strategies (*r* = *0.1*0) and positive attitude (*r* = *-0.0*5) were not significant. Thus, we excluded these variables from the model. Additionally, looking at the modification indices, we observed that two links were missing for the post-traumatic growth variables: with psychological inflexibility and with orientation to the problem. The model was modified accordingly generating the hypotheses detailed in Table 3, and then tested.

**Fig. 3:**
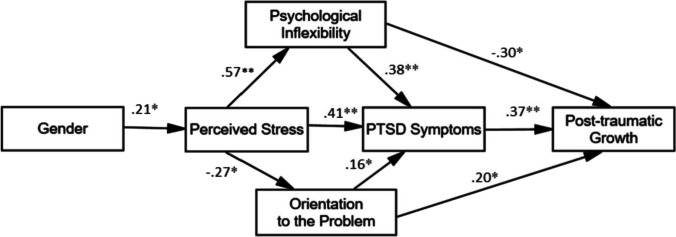
The path model illustrates direct and indirect effects and causal paths linking variables with Post-Traumatic Growth. *Note.* Values on the line = standardized path coefficient. * *p* < 0.05 and ** *p* < 0.01. Gender: F = 1 and M = 2

The fit indices for the model were excellent (CFI = 0.99, TLI = 0.97, RMSEA = 0.055 [90%CI = 0.001–0.144], and SRMS = 0.49. Estimates of the path coefficients and the results of significance testing are presented in Table 4 and Fig. 3. All the path coefficients were found to be statistically significant (Table 4). As for the exogenous variables, the standardized regression coefficient for gender predicted 21% of perceived stress variability. In turn, perceived stress explained orientation to the problem (27%) and psychological inflexibility (57%). Orientation to the problem and psychological inflexibility explained 16% and 38% of PTSD Symptoms, respectively. Finally, orientation to the problem, psychological inflexibility, and PTSD Symptoms explained 20%, 30%, and 37% of post-traumatic growth, respectively.

**Table 4:** Results from testing the hypotheses using path analysis parameter estimates

*Hypotheses*	*Path*	*Path coefficient [90%CI]*	*t*	*P*
H2	Gender → PSS-10	-0.21 [-0.34;-0.06]	-2.46	0.014
H3	PSS-10 → COPE-OP	-0.27 [-0.40;-0.14]	-3.19	0.001
H4	PSS-10 → AAQ-II	0.57 [0.48;0.65]	7.88	< 0.001
H5	PSS-10 → IES-R	0.41 [0.29;0.51]	5.01	< 0.001
H6	COPE-OP → IES-R	0.16 [0.04;0.27]	2.20	0.028
	COPE-OP → PTGI	0.20 [0.12;0.39]	2.26	0.024
H7	AAQ-II → IES-R	0.38 [0.25;0.51]	4.55	< 0.001
	AAQ-II → PTGI	-0.30 [-0.45;-0.13]	-2.84	0.004
H8	IES-R → PTGI	0.37 [0.22;0.57]	3.75	< 0.001

Gender: F = 1 and M = 2. PSS-10 = Perceived Stress Scale, AAQ-II = Acceptance and Action Questionnaire-II, COPE-AS = avoidance strategies, COPE-OP = orientation to the problem, COPE-PA = positive attitude, IES-R = Impact of Event Scale-Revised, PTGI = Post-Traumatic Growth Inventory

Concerning indirect effects, we observed the following ones. Gender had indirect effects on orientation to the problem (β = 0.06 [90%CI: 0.02;0.12]), psychological inflexibility (β = -0.12 [90%CI: -0.20;-0.04]), and PTSD Symptoms (β = -0.12 [90%CI: -0.20;-0.04]. Perceived Stress had an indirect effect on PTSD Symptoms (β = 0.18 [90%CI: 0.10;0.27]). Finally, both orientation to the problem (β = 0.06 [90%CI: 0.01;0.13]) and psychological inflexibility (β = 0.14 [90%CI: 0.08;0.26]) had an indirect effect on post-traumatic growth.

## Discussion

The aim of Study 2 was to investigate whether specific coping strategies and psychological inflexibility mediate the association between perceived stress and PTSD Symptoms during the first wave of COVID-19 Pandemic one year later. The analyses revealed that post-traumatic growth was negatively related to psychological inflexibility and positively related to PTSD symptoms and orientation to the problem, respectively. Moreover, PTSD symptoms were positively associated with post-traumatic growth one year later and orientation to the problem and psychological inflexibility affected the level of post-traumatic growth. These results support Solomon and Dekel’s (2007) findings that the ability to grow from traumatic stress does not only depend on the presence of traumatic symptoms, but other growth-related factors. Indeed, psychological inflexibility may restrict the amount of growth that one perceives while mindful awareness may provide opportunities to reignite a sense of resilience. These findings align with Boykin et al. (2020) who also found that psychological inflexibility may affect post-traumatic growth. Furthermore, orientation to the problem significantly affected PTSD symptoms and posttraumatic growth differentially, suggesting that it may be a short-term pain and long-term gain coping strategy.

## General discussion

The present study aimed to investigate the associations between perceived stress, posttraumatic stress, and posttraumatic growth with mediating variables of coping strategies and psychological inflexibility. Study 1 represents the first attempt to examine the association between perceived stress and PTSD symptoms and the mediating role of psychological inflexibility and coping strategies in this relationship, taking into account gender and COVID-19 stressful events. The analysis carried out confirmed the hypothetical-theoretical model. In particular, COVID-19 stressful events and female gender positively predict the levels of perceived stress. This finding is consistent with several studies. Salari et al. (2020) reported a high prevalence of perceived stress in the general population worldwide with the pandemic, with women reporting greater psychological distress than men. These results were further aligned with Rossi et al. (2020) and Mousavi et al. (2020), who underline the importance of the COVID-19 stressful events in predicting perceived stress. In particular, Rossi et al. (2020) found that having contracted the COVID-19 and having a loved one inflected or deceased due to COVID-19 heightened the levels of perceived stress. In the same manner, Mousavi et al. (2020) found that also having contracted the COVID-19 positively predict the levels of perceived stress.

Our data also showed that perceived stress is positively related to PTSD symptoms, which confirmed the finding of previous studies conducted in other contexts such as ongoing terrorist attacks (Besser et al., 2009) and oral cancer (Zhang et al., 2021). Ikizer et al. (2021) also found the same association in the context of the COVID-19 Pandemic, suggesting that this result could be explained by the limited access to psychological resources that generally help to cope with an unexpected aversive event. Thus, we decided to evaluate the mediating role of psychological inflexibility and different coping strategies on the relationship between perceived stress and PTSD symptoms during the first wave of COVID-19 Pandemic. Specifically, we found that psychological inflexibility was positively related to PTSD Symptoms, which is consistent with other evidence (Meyer et al., 2013; Plumb et al., 2004; Tull & Roemer, 2003). Due to the malleable nature of psychological inflexibility, it can represent a target for psychosocial interventions (Meyer et al., 2019).

In addition, our results suggest that also some coping strategies (i.e., avoidance strategies and orientation to the problem) were positively related to PTSD Symptoms. This finding was unexpected and may suggest the difficulty to establish which coping strategies might be considered adaptive or maladaptive in the context of the COVID-19 Pandemic. Indeed, the strategies normally considered to be more functional (i.e., social support) did not align with lowered stress in the context of first wave of COVID-19 Pandemic, underlining the importance of considering the situation and timing in which they acted to establish whether they are functional or not, according to other evidence (Fischer et al., 2021; Moritz et al., 2016; Vaughn-Coaxum et al., 2018). Increased levels of frustration were reported throughout the COVID-19 Pandemic (Anjum et al., 2020), which seems to predict the level of perceived stress (Bardeen et al., 2017).

The aim of study 2 was to investigate whether psychological inflexibility and the use of some coping strategies mediate the relationship between perceived stress and PTSD symptoms including post-traumatic growth one-year later. The analyses demonstrated that post-traumatic growth was negatively related to psychological inflexibility and positively related to PTSD symptoms and orientation to the problem, respectively. Interestingly, orientation to the problem positively predicted PTSD symptoms and post-traumatic growth one year later. Orientation strategies involve the facilitation of approach-oriented problem-solving, which involves comprehensive analysis of the current situation and ultimately, a lack of avoidance. Thus, although orientation to the problem may predict greater symptoms in the short term, it may produce post-traumatic growth in the long-term. The positive relationship between orientation to the problem and post-traumatic growth has also been found in previous literature, specifically in cancer patients (Markman et al., 2020; Widows et al., 2005) and in veterans with lower-limb amputations (Tuncay & Musabak, 2015). Researchers have quoted that *“Individuals with a positive problem orientation believe that problems are challenges to be solved rather than threats”* (Adrian et al., 2010, p. 328). In other words, perceiving oneself as being proactive and able to find a positive solution to a traumatic experience may promote post-traumatic growth in order to evolve after the traumatic event and not simply recover from it (Long & Gallagher, 2017).

In contrary, psychological inflexibility is positively associated with more PTSD symptoms in the short term and less post-traumatic growth in the long-term. Thus, comparatively, it appears that orientation to the problem may be an adaptive strategy in the long term and psychological inflexibility may not have any long-term benefits.

The relationship between PTSD Symptoms and post-traumatic growth has been largely documented in previous studies (Koliouli & Canellopoulos, 2021; Solomon & Dekel, 2007), which suggested that post-traumatic growth promotes a cognitive adaptation process through positive reframing of the traumatic event (Koliouli & Canellopoulos, 2021; Solomon & Dekel, 2007). In line with our results, Misurya et al. (2021) found that psychological flexibility modulates post-traumatic growth. In other words, being psychologically flexible facilitates acceptance and understanding of the emotional aspects related to the traumatic event, thus promoting post-traumatic growth. Therefore, we may hypothesize that the presence of low psychological flexibility (i.e., psychological inflexibility) limits acceptance and understanding, thus reducing the development of post-traumatic growth in the context of the COVID-19 Pandemic.

### The theoretical and practical contribution of the study

In this study, particular emphasis was placed on the strategies that can help individuals to modulate (i.e., increase or decrease) the association between perceived stress and PTSD symptoms, as well as to increase post-traumatic growth one year later. As regards the implications for clinical practice, our results suggest the usefulness of preferring therapeutic approaches related to the third-wave psychological interventions of Cognitive Behavioral Therapy (CBT; Beck, 1976), such as Acceptance Commitment Therapy (ACT; Hayes et al., 1999) and Mindfulness interventions (Halland et al., 2015; Kabat-Zinn, 1990).In particular, ACT places psychological inflexibility as the essential direct target of treatment (Hayes et al., 1999; Krafft et al., 2018): therefore, using ACT interventions to teach psychological flexibility techniques may help individuals to reduce the impacts of stress (Hayes et al., 2006; Tavakoli et al., 2019), even related to the COVID-19 Pandemic (Arslan et al., 2021; Dawson & Golijani-Moghaddam, 2020). There is certainly a link between psychological flexibility and coping strategies (e.g., orientation to the problem) (Dawson & Golijani-Moghaddam, 2020). For this reason, we believe it is essential to implement ACT-based treatments, which promote psychological flexibility, in order to overcome stress-related difficulties and encourage healthy personal growth. Mindfulness training may help to process stressful events into less complicated challenges, by promoting orientation to the problem approaches (Halland et al., 2015). Future studies may investigate whether mindfulness may help individuals cope with stressful events related to the COVID-19 Pandemic (Accoto et al., 2021; Weis et al., 2021).

### Limitations

This study has some limitations. First, the sampling method is not true random sampling, given that recruitment was on social media and may restrict access to those who are not connected to social media. Second, recent studies have found that centrality, the degree to which an individual perceives a distressful event as central to one’s identity and worldviews, affect interindividual and intraindividual differences in coping strategies and experience of post-traumatic growth (Boykin & Teng, 2019). Thus, future studies should evaluate how impactful participants believed COVID-19 to have shaped their identity and life experiences. Third, self-reported measures were administered to assess the dimensions of this study. Future research should employ different methods (e.g., clinician-ratings, peer-ratings) to reduce self-report biases. Finally, the COVID-19 stressful events measures may not comprehensively cover all possible stressful events (e.g., loss of a job, financial burden, increase in grocery prices, problems with working from home, lack of pleasure from going to restaurants and travelling). Future studies should examine a more comprehensive list of stressful events.

In light of our results, we suggest implementing interventions, such as ACT and mindfulness training, that target psychological inflexibility and coping strategies, thus reducing the association between perceived stress and PTSD symptoms and increasing post-traumatic growth.

## Conclusion

In sum, our results suggest that the association between perceived stress and PTSD symptoms is also present in the context of COVID-19 Pandemic, particularly among women. Both adaptive (e.g., orientation to the problem) and maladaptive coping strategies (e.g., avoidance strategies) and psychological inflexibility are positively related with PTSD Symptoms.

Moreover, orientation to the problem explains mostly PTSD symptoms expression compared to avoidance strategies. Indeed, in the long term (i.e., a year later), people who used coping strategies generally considered adaptive (e.g., orientation to the problem) experienced greater post-traumatic growth than those with greater levels of psychological inflexibility. This underlines that an increase in symptomology may not be maladaptive if the psychological growth that comes with it is taken into context.

## Data Availability

All data and materials are available from the first author upon request.

## References

[CR1] Accoto, A., Chiarella, S. G., Raffone, A., Montano, A., de Marco, A., Mainiero, F., ... & Conversi, D. (2021). Beneficial effects of mindfulness-based stress reduction training on the well-being of a female sample during the first total lockdown due to Covid-19 pandemic in Italy. *International journal of environmental research and public health*, *18*(11), 5512. 10.3390/ijerph18115512 10.3390/ijerph18115512 PMC8196575 34063864

[CR2] Adrian M, Lyon AR, Oti R, Tininenko J (2010). Developmental foundations and clinical applications of social information processing: A review. Marriage & Family Review.

[CR3] American Psychiatric Association (2013). Diagnostic and Statistical Manual of Mental Disorders, Fifth Edition: (DSM-5).

[CR4] Anjum, S., Ullah, R., Rana, M. S., Ali Khan, H., Memon, F. S., Ahmed, Y., ... & Faryal, R. (2020). COVID-19 pandemic: A serious threat for public mental health globally. *Psychiatria Danubina*, *32*(2), 245–250. 10.24869/psyd.2020.245 10.24869/psyd.2020.245 32796793

[CR5] Arslan G, Yıldırım M, Tanhan A, Buluş M, Allen KA (2021). Coronavirus stress, optimism-pessimism, psychological inflexibility, and psychological health: Psychometric properties of the Coronavirus Stress Measure. International Journal of Mental Health and Addiction.

[CR6] Bandalos DL (2014). Relative performance of categorical diagonally weighted least squares and robust maximum likelihood estimation. Structural Equation Modeling: A Multidisciplinary Journal.

[CR7] Bardeen JR, Fergus TA (2016). The interactive effect of cognitive fusion and experiential avoidance on anxiety, depression, stress and posttraumatic stress symptoms. Journal of Contextual Behavioral Science.

[CR8] Bardeen JR, Fergus TA, Orcutt HK (2017). Examining the specific dimensions of distress tolerance that prospectively predict perceived stress. Cognitive Behaviour Therapy.

[CR9] Beck AT (1976). Cognitive therapy and the emotional disorders.

[CR10] Besser A, Neria Y, Haynes M (2009). Adult attachment, perceived stress, and PTSD among civilians exposed to ongoing terrorist attacks in Southern Israel. Personality and Individual Differences.

[CR11] Bond, F. W., Hayes, S. C., Baer, R. A., Carpenter, K. M., Guenole, N., Orcutt, H. K., ... & Zettle, R. D. (2011). Preliminary psychometric properties of the Acceptance and Action Questionnaire–II: A revised measure of psychological inflexibility and experiential avoidance. *Behavior Therapy, 42*(4), 676–688. 10.1016/j.beth.2011.03.007 22035996

[CR12] Bovier PA, Chamot E, Perneger TV (2004). Perceived stress, internal resources, and social support as determinants of mental health among young adults. Quality of Life Research.

[CR13] Boykin, D. M., & Teng, E. J. (2019). A proposal for augmenting the measurement of index events in PTSD assessment using event centrality. *Anxiety, Stress, & Coping, 32*(5), 559–567. 10.1080/10615806.2019.1638682 31272207

[CR14] Boykin DM, Anyanwu J, Calvin K, Orcutt HK (2020). The moderating effect of psychological flexibility on event centrality in determining trauma outcomes. Psychological Trauma: Theory, Research, Practice, and Policy.

[CR15] Bridgland, V. M., Moeck, E. K., Green, D. M., Swain, T. L., Nayda, D. M., Matson, L. A., ... & Takarangi, M. K. (2021). Why the COVID-19 pandemic is a traumatic stressor. *PloS one*, 16(1), e0240146. 10.1371/journal.pone.0240146 10.1371/journal.pone.0240146 PMC7799777 33428630

[CR16] Brockman, C., Snyder, J., Gewirtz, A., Gird, S. R., Quattlebaum, J., Schmidt, N., ... & DeGarmo, D. (2016). Relationship of service members’ deployment trauma, PTSD symptoms, and experiential avoidance to postdeployment family reengagement. *Journal of Family Psychology*, *30*(1), 52. 10.1037/fam0000152 10.1037/fam0000152 PMC4804869 26437144

[CR17] Cai H, Tu B, Ma J, Chen L, Fu L, Jiang Y, Zhuang Q (2020). Psychological impact and coping strategies of frontline medical staff in Hunan between January and March 2020 during the outbreak of coronavirus disease 2019 (COVID-19) in Hubei, China. Medical Science Monitor: International Medical Journal of Experimental and Clinical Research.

[CR18] Chao RCL (2012). Managing perceived stress among college students: The roles of social support and dysfunctional coping. Journal of College Counseling.

[CR19] Chrousos GP (2009). Stress and disorders of the stress system. Nature Reviews Endocrinology.

[CR20] Clohessy S, Ehlers A (1999). PTSD symptoms, response to intrusive memories and coping in ambulance service workers. British Journal of Clinical Psychology.

[CR21] Cofini V, Carbonelli A, Cecilia MR, Binkin N, di Orio F (2015). Post traumatic stress disorder and coping in a sample of adult survivors of the Italian earthquake. Psychiatry Research.

[CR22] Coroiu A, Körner A, Burke S, Meterissian S, Sabiston CM (2016). Stress and posttraumatic growth among survivors of breast cancer: A test of curvilinear effects. International Journal of Stress Management.

[CR23] Crabtree MA, Hale WJ, Meyer EC, Kimbrel NA, DeBeer BB, Gulliver SB, Morissette SB (2021). Dynamics of risk: Recent changes in psychological inflexibility precede subsequent changes in returning US veterans' posttraumatic stress. Journal of Clinical Psychology.

[CR24] Dawson DL, Golijani-Moghaddam N (2020). COVID-19: Psychological flexibility, coping, mental health, and wellbeing in the UK during the pandemic. Journal of Contextual Behavioral Science.

[CR25] Fischer R, Scheunemann J, Moritz S (2021). Coping strategies and subjective well-being: Context matters. Journal of Happiness Studies.

[CR26] Flesia L, Monaro M, Mazza C, Fietta V, Colicino E, Segatto B, Roma P (2020). Predicting perceived stress related to the Covid-19 outbreak through stable psychological traits and machine learning models. Journal of Clinical Medicine.

[CR27] Foà C, Tonarelli A, Caricati L, Fruggeri L (2015). COPE-NVI-25: Validazione italiana della versione ridotta della Coping Orientation to the Problems Experienced (COPE-NVI). Psicologia della salute.

[CR28] Folkman S, Moskowitz JT (2004). Coping: Pitfalls and promise. Annual Review of Psychology.

[CR29] Folkman, S. (2010). 22 Stress, health, and coping: Synthesis, commentary, and future directions. *The Oxford handbook of stress, health, and coping*, 453.

[CR30] Gan Y, Fu Q (2022). Risk perception and coping response to COVID-19 mediated by positive and negative emotions: A study on Chinese college students. PLoS ONE.

[CR31] Gan Y, Zhang J, Quan Z (2021). Public perception of risk and coping response to COVID-19 in China: The moderating role of negative emotion. Journal of Psychology in Africa.

[CR32] Groarke A, Curtis R, Groarke JM, Hogan MJ, Gibbons A, Kerin M (2017). Post-traumatic growth in breast cancer: How and when do distress and stress contribute?. Psycho-Oncology.

[CR33] Grosso JA, Kimbrel NA, Dolan S, Meyer EC, Kruse MI, Gulliver SB, Morissette SB (2014). A test of whether coping styles moderate the effect of PTSD symptoms on alcohol outcomes. Journal of Traumatic Stress.

[CR34] Gutner CA, Rizvi SL, Monson CM, Resick PA (2006). Changes in coping strategies, relationship to the perpetrator, and posttraumatic distress in female crime victims. Journal of Traumatic Stress: Official Publication of the International Society for Traumatic Stress Studies.

[CR35] Halland, E., De Vibe, M., Solhaug, I., Friborg, O., Rosenvinge, J. H., Tyssen, R., ... & Bjørndal, A. (2015). Mindfulness training improves problem-focused coping in psychology and medical students: Results from a randomized controlled trial. *College Student Journal*, *49*(3), 387-398.

[CR36] Hayes SC, Strosahl KD, Wilson KG (1999). Acceptance and commitment therapy: An experiential approach to behavior change.

[CR37] Hayes SC, Luoma JB, Bond FW, Masuda A, Lillis J (2006). Acceptance and commitment therapy: Model, processes and outcomes. Behaviour Research and Therapy.

[CR38] Hayes SC, Strosahl KD, Wilson KG (2011). Acceptance and commitment therapy: The process and practice of mindful change.

[CR39] Hooberman J, Rosenfeld B, Rasmussen A, Keller A (2010). Resilience in trauma-exposed refugees: The moderating effect of coping style on resilience variables. American Journal of Orthopsychiatry.

[CR40] Huang C, Xie J, Owusua T, Chen Z, Wang J, Qin C, He Q (2021). Is psychological flexibility a mediator between perceived stress and general anxiety or depression among suspected patients of the 2019 coronavirus disease (COVID-19)?. Personality and Individual Differences.

[CR41] Ikizer G, Karanci AN, Gul E, Dilekler I (2021). Post-traumatic stress, growth, and depreciation during the COVID-19 pandemic: Evidence from Turkey. European Journal of Psychotraumatology.

[CR42] Jackson DL (2001). Sample size and number of parameter estimates in maximum likelihood confirmatory factor analysis: A Monte Carlo investigation. Structural Equation Modeling.

[CR43] Kabat-Zinn J (1990). Full catastrophe living: Using the wisdom of your body and mind to face stress, pain, and illness.

[CR44] Kashdan TB, Morina N, Priebe S (2009). Post-traumatic stress disorder, social anxiety disorder, and depression in survivors of the Kosovo War: Experiential avoidance as a contributor to distress and quality of life. Journal of Anxiety Disorders.

[CR45] Kennedy C, Deane FP, Chan AY (2021). Intolerance of uncertainty and psychological symptoms among people with a missing loved one: Emotion regulation difficulties and psychological inflexibility as mediators. Journal of Contextual Behavioral Science.

[CR46] Khalid I, Khalid TJ, Qabajah MR, Barnard AG, Qushmaq IA (2016). Healthcare workers emotions, perceived stressors and coping strategies during a MERS-CoV outbreak. Clinical Medicine & Research.

[CR47] Koliouli F, Canellopoulos L (2021). Dispositional Optimism, Stress, Post-traumatic stress Disorder and Post-traumatic Growth in Greek general population facing the COVID-19 crisis. European Journal of Trauma & Dissociation.

[CR48] Krafft J, Ferrell J, Levin ME, Twohig MP (2018). Psychological inflexibility and stigma: A meta-analytic review. Journal of Contextual Behavioral Science.

[CR49] Krishnamoorthy Y, Nagarajan R, Saya GK, Menon V (2020). Prevalence of psychological morbidities among general population, healthcare workers and COVID-19 patients amidst the COVID-19 pandemic: A systematic review and meta-analysis. Psychiatry Research.

[CR50] Levin ME, MacLane C, Daflos S, Seeley JR, Hayes SC, Biglan A, Pistorello J (2014). Examining psychological inflexibility as a transdiagnostic process across psychological disorders. Journal of Contextual Behavioral Science.

[CR51] Linley PA, Joseph S (2004). Positive change following trauma and adversity: A review. Journal of Traumatic Stress: Official Publication of the International Society for Traumatic Stress Studies.

[CR52] Long, L. J., & Gallagher, M. W. (2017). *Hope and Post-Traumatic Stress Disorder. The Oxford Handbook of Hope*.

[CR53] Markman ES, McClure KS, McMahon CE, Zelikovsky N, Macone BW, Bullock AJ (2020). Social problem solving and posttraumatic growth new possibilities in postoperative breast cancer survivors. Journal of Clinical Psychology in Medical Settings.

[CR54] Meyer EC, Morissette SB, Kimbrel NA, Kruse MI, Gulliver SB (2013). Acceptance and Action Questionnaire—II scores as a predictor of posttraumatic stress disorder symptoms among war veterans. Psychological Trauma: Theory, Research, Practice, and Policy.

[CR55] Meyer EC, La Bash H, DeBeer BB, Kimbrel NA, Gulliver SB, Morissette SB (2019). Psychological inflexibility predicts PTSD symptom severity in war veterans after accounting for established PTSD risk factors and personality. Psychological Trauma: Theory, Research, Practice, and Policy.

[CR56] Misurya, P., Shukla, V., & Anand, P. V. (2021). Self-Compassion and Post Traumatic Growth: The Mediating Role of Psychological Flexibility. 10.31234/osf.io/q5fjd

[CR57] Mondo M, Sechi C, Cabras C (2019). Psychometric evaluation of three versions of the Italian Perceived Stress Scale. Current Psychology.

[CR58] Moritz S, Jahns AK, Schröder J, Berger T, Lincoln TM, Klein JP, Göritz AS (2016). More adaptive versus less maladaptive coping: What is more predictive of symptom severity? Development of a new scale to investigate coping profiles across different psychopathological syndromes. Journal of Affective Disorders.

[CR59] Motta Zanin G, Gentile E, Parisi A, Spasiano D (2020). A preliminary evaluation of the public risk perception related to the COVID-19 health emergency in Italy. International Journal of Environmental Research and Public Health.

[CR60] Mousavi SAM, Hooshyari Z, Ahmadi A (2020). The most stressful events during the COVID-19 epidemic. Iranian Journal of Psychiatry.

[CR61] Musazzi L, Tornese P, Sala N, Popoli M (2017). Acute stress is not acute: Sustained enhancement of glutamate release after acute stress involves readily releasable pool size and synapsin I activation. Molecular Psychiatry.

[CR62] Ng, M. Y., Lee, E. Y., Yang, J., Yang, F., Li, X., Wang, H., ... & Kuo, M. D. (2020). Imaging profile of the COVID-19 infection: radiologic findings and literature review. *Radiology: Cardiothoracic Imaging*, *2*(1), e200034. 10.1148/ryct.2020200034 10.1148/ryct.2020200034 PMC7233595 33778547

[CR63] Ozer EJ, Best SR, Lipsey TL, Weiss DS (2003). Predictors of posttraumatic stress disorder and symptoms in adults: A meta-analysis. Psychological Bulletin.

[CR64] Pachter, L. M., Bernstein, B. A., Szalacha, L., & Coll, C. G. (2013, July). Racism in minority youth: the relationship between emotional and coping responses and behavioral health. In *journal of developmental and behavioral pediatrics* (Vol. 34, No. 6, pp. S3-S3). 530 walnut st, philadelphia, pa 19106–3621 usa: lippincott williams & wilkins.

[CR65] Pakenham KI, Landi G, Boccolini G, Furlani A, Grandi S, Tossani E (2020). The moderating roles of psychological flexibility and inflexibility on the mental health impacts of COVID-19 pandemic and lockdown in Italy. Journal of Contextual Behavioral Science.

[CR66] Pennato T, Berrocal C, Bernini O, Rivas T (2013). Italian version of the acceptance and action questionnaire-II (AAQ-II): Dimensionality, reliability, convergent and criterion validity. Journal of Psychopathology and Behavioral Assessment.

[CR67] Phua DH, Tang HK, Tham KY (2005). Coping responses of emergency physicians and nurses to the 2003 severe acute respiratory syndrome outbreak. Academic Emergency Medicine.

[CR68] Pietrantonio F, De Gennaro L, Di Paolo MC, Solano L (2003). The impact of event scale: Validation of an Italian version. Journal of Psychosomatic Research.

[CR69] Plumb JC, Orsillo SM, Luterek JA (2004). A preliminary test of the role of experiential avoidance in post-event functioning. Journal of Behavior Therapy and Experimental Psychiatry.

[CR70] Prati G, Pietrantoni L (2014). Italian adaptation and confirmatory factor analysis of the full and the short form of the Posttraumatic Growth Inventory. Journal of Loss and Trauma.

[CR71] Rossi, R., Socci, V., Talevi, D., Mensi, S., Niolu, C., Pacitti, F., ... & Di Lorenzo, G. (2020). COVID-19 pandemic and lockdown measures impact on mental health among the general population in Italy. *Frontiers in Psychiatry*, 790. 10.3389/fpsyt.2020.00790 10.3389/fpsyt.2020.00790 PMC7426501 32848952

[CR72] Salari, N., Hosseinian-Far, A., Jalali, R., Vaisi-Raygani, A., Rasoulpoor, S., Mohammadi, M., ... & Khaledi-Paveh, B. (2020). Prevalence of stress, anxiety, depression among the general population during the COVID-19 pandemic: a systematic review and meta-analysis. *Globalization and Health*, *16*(1), 1-11. 10.1186/s12992-020-00589-w 10.1186/s12992-020-00589-w PMC7338126 32631403

[CR73] Schramm AT, Pandya K, Fairchild AJ, Venta AC, deRoon-Cassini TA, Sharp C (2020). Decreases in psychological inflexibility predict PTSD symptom improvement in inpatient adolescents. Journal of Contextual Behavioral Science.

[CR74] Schreiber JB, Nora A, Stage FK, Barlow EA, King J (2006). Reporting structural equation modeling and confirmatory factor analysis results: A review. The Journal of Educational Research.

[CR75] Solomon Z, Dekel R (2007). Posttraumatic stress disorder and posttraumatic growth among Israeli ex-pows. Journal of Traumatic Stress: Official Publication of the International Society for Traumatic Stress Studies.

[CR76] Taku K, Cann A, Tedeschi RG, Calhoun LG (2015). Core beliefs shaken by an earthquake correlate with posttraumatic growth. Psychological Trauma: Theory, Research, Practice, and Policy.

[CR77] Tavakoli N, Broyles A, Reid EK, Sandoval JR, Correa-Fernández V (2019). Psychological inflexibility as it relates to stress, worry, generalized anxiety, and somatization in an ethnically diverse sample of college students. Journal of Contextual Behavioral Science.

[CR78] Tedeschi RG, Calhoun LG (2004). Posttraumatic growth: Conceptual foundations and empirical evidence. Psychological Inquiry.

[CR79] Tull MT, Roemer L (2003). Alternative explanations of emotional numbing of posttraumatic stress disorder: An examination of hyperarol and experiential avoidance. Journal of Psychopathology and Behavioral Assessment.

[CR80] Tuncay T, Musabak I (2015). Problem-focused coping strategies predict posttraumatic growth in veterans with lower-limb amputations. Journal of Social Service Research.

[CR81] Vaughn-Coaxum RA, Wang Y, Kiely J, Weisz JR, Dunn EC (2018). Associations between trauma type, timing, and accumulation on current coping behaviors in adolescents: Results from a large, population-based sample. Journal of Youth and Adolescence.

[CR82] Vindegaard N, Benros ME (2020). COVID-19 pandemic and mental health consequences: Systematic review of the current evidence. Brain, Behavior, and Immunity.

[CR83] Weis R, Ray SD, Cohen TA (2021). Mindfulness as a way to cope with COVID-19-related stress and anxiety. Counselling and Psychotherapy Research.

[CR84] Widows MR, Jacobsen PB, Booth-Jones M, Fields KK (2005). Predictors of posttraumatic growth following bone marrow transplantation for cancer. Health Psychology.

[CR85] Wolf EJ, Harrington KM, Clark SL, Miller MW (2013). Sample size requirements for structural equation models: An evaluation of power, bias, and solution propriety. Educational and Psychological Measurement.

[CR86] Xu J, Wu W (2014). Work satisfaction and posttraumatic growth 1 year after the 2008 Wenchuan earthquake: The perceived stress as a moderating factor. Archives of Psychiatric Nursing.

[CR87] Yeung NC, Lu Q (2018). Perceived stress as a mediator between social support and posttraumatic growth among Chinese American breast cancer survivors. Cancer Nursing.

[CR88] Zhang Y, Cui C, Wang L, Yu X, Wang Y, Wang X (2021). The mediating role of hope in the relationship between perceived stress and post-traumatic stress disorder among Chinese patients with oral cancer: A cross-sectional study. Cancer Management and Research.

